# Determining the Impact of Lifestyle on the Health of Primary School Children in Slovenia Through Mixed Membership Focus Groups

**DOI:** 10.1007/s10900-023-01231-7

**Published:** 2023-05-09

**Authors:** Rebeka Lekše, Dijana Godec, Mirko Prosen

**Affiliations:** 1 Faculty of Health Sciences, Department of Nursing, University of Primorska, Polje 42, Izola, 6310 Slovenia; 2 Health Promotion Centre, Gregorčičeva cesta 8, Ilirska Bistrica, 6250 Slovenia

**Keywords:** Children’s health, health promotion, community involvement, health education

## Abstract

Lifestyle choices made by children and adolescents can lead to a variety of health problems. It is therefore crucial to evaluate the state health-related lifestyles of primary school children’s and identify areas for improvement. The aim of this study was to investigate the experiences and perspectives of those involved in educating current generations of primary school children about their lifestyle and the associated health implications of their behavior. Four focus groups were formed, comprising parents, primary school teachers, health professionals, and leisure activity teachers, with five participants in each group. Semi-structured interviews were conducted with each group as part of the qualitative research paradigm of the study. The study revealed three central themes, emphasizing the need to promote health and educate children about healthy behaviors, environmental influences, and health literacy. Community nurses need to take a proactive role in promoting school health. Although adults, peers, and the environment exert a strong influence on children’s lifestyles, they often fail to live up to their responsibilities and allow harmful habits to form. To instill positive behaviors and an optimistic attitude towards a healthy lifestyle, all those involved in a child’s education need to collaborate. Health-related topics should be included or reinforced in primary school curricula, and parents and school staff should receive appropriate training.

**Supplementary Information:**

The online version contains supplementary material available at 10.1007/s10900-023-01231-7.

## Introduction

The term lifestyle refers to a set of behaviors that an individual engages in over a period of time, which may include both health-promoting and health-damaging practices [38]. A healthy lifestyle is crucial not only for health indicators in the narrow sense, but also for economic, social and other indicators as health also forms the foundation for learning, work, civic activities, and performance [34, 54].

The fact that childhood habits often persist into adulthood accentuates the need to promote healthy practices early on. Due to increased unhealthy behaviors such as poor diet, physical inactivity, and smoking, there has been an increase in lifestyle-related health problems such as obesity, heart disease, and diabetes [56]. Children, who are still developing physically, mentally, and emotionally, are particularly vulnerable to these effects [66], which further emphasizes the importance of instilling healthy habits at a young age to ensure proper growth and development. Moreover, adopting healthy behaviors can improve academic performance, self-esteem, and reduce mental health problems [60, 82].

Children’s lifestyles are shaped by a complex interplay of determinants, including social, environmental, cultural, and economic factors [3, 31, 54]. These determinants play a crucial role in the development of healthy or unhealthy behaviors and lifestyle habits, and have long-term effects for children’s health and well-being. A child’s lifestyle can be impacted by social factors, such as family structure and access to health care, and environmental factors, such as availability of healthy food options and exposure to environmental pollutants. Cultural determinants such as health-related beliefs and economic determinants such as income can also influence children’s access to resources for healthy living. In this regard, positive determinants such as safe parks and a supportive family environment can lead to better physical and mental health outcomes, while negative determinants such as exposure to air pollution and negative social norms can lead to various health problems [9, 25].

To identify areas where efforts should be directed for effective intervention, global monitoring of lifestyle trends and patterns in children is crucial. Regular assessments conducted by the World Health Organization (WHO) [77] valuable insights into the prevalence of health behaviors such as physical activity, physical inactivity, and healthy eating habits [37, 78]. As concerns over escalating global prevalence rates of childhood obesity persistently mount concurrent with adverse associated health outcomes [27, 78], consequently questioning current lifestyles’ impact on well-being. Exploring children’s lifestyle patterns and trends through diverse cultures is imperative to develop effective interventions for ameliorating children’s health outcomes. A comprehensive analysis of worldwide research on children’s lifestyles proves instrumental in revealing the prospects and challenges encountered by promoting healthful behaviors while reducing childhood illnesses’ prevalence.

Research conducted to date has shown that parents, teachers, peers, and health professionals play a critical role in shaping a child’s lifestyle. Creating an environment conducive to healthy habits within the home is one of the many ways parents can encourage healthy behaviors among their children [13]. Teachers can incorporate physical activity and healthy eating habits into the school curriculum, while peers can influence a child’s behavior through social norms and peer pressure [54]. Health professionals can provide parents and children with guidance and support concerning healthy behaviors and lifestyle changes [39]. Despite the efforts of various stakeholders, unhealthy behaviors and lifestyle-related health problems among children remain a major challenge [6]. Various interventions have already been implemented; however, their effectiveness and long-term sustainability remain unclear.

Maintaining positive lifestyle patterns among children entails adopting an all-inclusive approach involving such as parents or guardians, primary school instructors as well as healthcare professionals. Promoting activities such as healthy eating habits incorporation of regular exercise routines into daily regimens alongside adequate rest periods are mechanisms through which optimal life quality can be achieved by ensuring disease prevention [2]. It is therefore important to understand the behaviors and patterns influencing children’s lifestyles and health, especially during their primary school years [71]. Such understanding can inform efforts aimed at promoting healthy behaviors and preventing the development of unhealthy habits that can cause long-term health problems. Health-promoting behaviors, i.e. multidimensional patterns of self-initiated actions and perceptions that serve to maintain or enhance an individual’s well-being, self-actualization and fulfilment, are viewed as actions taken by the individual to achieve positive outcomes [46].

The aim of this study was to explore how parents, teachers, community nurses, and leisure activity teachers perceive and understand primary school children’s health-related lifestyles through their own experiences, and how they view their role in the context of the development of children’s health literacy. To this end, the following research questions were formulated: »How do parents, community nurses, and school and leisure activity teachers experience, perceive, and understand primary school children’s health-related lifestyle behaviors and community influences which shape them?« and »How do parents, community nurses, and school and leisure activity teachers perceive their role in health promotion?«.

## Methods

The qualitative methodology employed a descriptive interpretive approach to understand the participants’ experiences, values, beliefs, and behaviors in relation to the context in which the research was conducted [23, 73]. Interpretive description is unique in that it recognizes that human experiences comprise complex interactions between psychosocial and biological phenomena [74]. Moreover, it is amenable to a re-evaluation in the light of differing contexts, concepts, and analytical frameworks [74].

### Participants

A purposive sample of parents, community nurses, and school and leisure activity teachers was recruited from a local community in south-western Slovenia. These groups were identified as important stakeholders who could influence the health behavior of primary school children. The final sample consisted of 20 participants divided into four focus groups (FGs) with different stakeholders. In determining the sample size, the power information criterion was taken into account, which states that the more relevant the information in the sample is to the chosen research, the less information and the fewer participants are needed [48]. In addition, three FGs were deemed sufficient to identify the predominant themes in the data set [29]. Teachers and nurses who had at least 5 years of professional experience in the field of child education and daily social interaction with children were eligible to participate.

### Data Collection

Data were collected using the focus group method. As suggested by Braun & Clarke [17], the FG guiding questions were formulated based on a literature review [7, 57, 65, 70] and focused on the main research questions. The topic guide contained key themes or predetermined guiding questions related to the phenomenon under study (Table 1). Participants were informed in advance about the purpose and objectives of the study. FG sessions were conducted in the spring of 2022 at the participants’ workplace or at the school premises, with permission from the institution’s supervisors. A quiet room, free from major distractions, was provided for each FG session.

**Table 1:** Examples of questions included in the semi-structured interview

Question number	Questions from the semi-structured interview
1.	How do you experience and perceive the development of primary school children’s lifestyle and its impact on them?
1a	How do you perceive the lifestyle of children today (diet, exercise, habits, etc.)?
1b	What are the most common changes you have observed in children (closing down, irritability, fatigue, lack of self-confidence, etc.)?
1c	What are the most important factors that shape the lifestyle behaviors of primary school children?
1d	How do parents, teachers, and peers influence the lifestyle behaviors of primary school children?
1e	What are the challenges and barriers to promoting healthy lifestyle behaviors in primary school children?
1f	How do these changes affect the immediate environment (family, school, friends)?
1 g	What advantages and disadvantages has modern life brought to the development of children’s lifestyles?
2.	How do you see your role in promoting health and thereby shaping children’s lifestyles?
2a	How do you see your role in shaping children’s lifestyle?
2b	What do health promotion contents and activities mean to you?
2c	Do you believe you possess enough experience, skills, or knowledge in this area, or where do you obtain your knowledge?
2d	What are the key health promotion messages and strategies for promoting healthy lifestyles in children? What are the best practices for promoting healthy lifestyles in your particular field or profession?
2e	How can you collaborate with other professionals and stakeholders to promote healthy lifestyles in children?
2f	What are the challenges and barriers to promoting healthy lifestyles, and how can you overcome them?

Before the start of each FG session, participants were asked to provide general information, i.e. their age, number of years of work experience, and their occupation. All participants were informed that they could withdraw from the FG at any time without consequences. All FG sessions were audio-recorded, with one researcher responsible for leading the session and another for recording and taking notes. Each FG session was followed by a reflective discussion among the moderators, in which general impressions were recorded to facilitate subsequent analysis.

### Data Analysis

For the analysis of FG data, this study used the qualitative content analysis approach proposed by Graneheim [28]. Given that all FGs were conducted in Slovenian, this was also the language used in the data analysis. Initially, two researchers carefully read through each transcript to obtain a comprehensive understanding of the statements made in each group. Subsequently, after a line-by-line reading, meaning units were identified, condensed and assigned codes. In the next step, subcategories were labeled with codes based on similarities and differences. Similar subcategories were then used to extract categories from which research themes emerged. The categories and subcategories were discussed and assessed by all three team members [24, 67]. To ensure confirmability of the data analysis, a representative from each stakeholder group was consulted to provide feedback on the findings. The study was reported according to the consolidated criteria for presenting qualitative research (COREQ) [69, 75].

### Rigor and Trustworthiness

We followed the trustworthiness standards for qualitative research proposed by Lincoln and Guba [44].

To ensure the credibility of the study, a semi-structured interview guide was developed based on a literature review and consultation with experts in the field. To ensure transferability, the authors used a purposive sample, selecting FG participants who could offer a range of perspectives and experiences related to child health promotion. To ensure dependability, the authors employed a standardized approach to data collection and analysis that included audio recordings of the FG sessions and independent coding of the collected data by two researchers. Finally, to ensure confirmability, the authors maintained an audit trail for all data and analysis processes, and conducted continuous reflection to minimize bias, thus ensuring that the resulting intervention was grounded in the perspectives and experiences of the FG participants. The authors conclude that using FGs and adhering to the standards of trustworthiness proposed by Lincoln and Guba [44] was an effective approach to developing an evidence-based child health promotion intervention that meets the needs and experiences of the target population.

### Ethical Considerations

Participation in the study was voluntary and anonymous. To comply with the ethical considerations of the research, all participants in the study signed an informed consent form before starting the FG, thus familiarizing themselves with the data protection procedures, as well as the purpose and conduct of the research. All participants were also given the opportunity to receive the results of the study. The study was given ethical approval by the Institutional Review Board and conducted in accordance with the Helsinki Declaration.

## Results

A total of 20 participants took part in the study, including 16 women and 4 men, with an average age of 47.8 years and an average work experience of 26.1 years. The participants’ characteristics are presented in Table 2.

**Table 2:** Demographic characteristics of the participants (n = 20)

Number of the focus group	Gender	Age	Years of employment	Code name
Focus Group 1 (Healthcare workers)	Female	63	35	Health Professional 1
	Female	50	32	Health Professional 2
	Female	48	27	Health Professional 3
	Female	39	18	Health Professional 4
	Female	49	31	Health Professional 5
Focus Group 2 (Primary school teachers)	Female	50	29	Teacher 1
	Male	42	21	Teacher 2
	Female	56	38	Teacher 3
	Female	47	26	Teacher 4
	Female	61	43	Teacher 5
Focus Group 3 (Leisure activity teachers)	Male	57	35	Leisure Activity Teacher 1
	Female	35	13	Leisure Activity Teacher 2
	Female	63	38	Leisure Activity Teacher 3
	Female	43	21	Leisure Activity Teacher 4
	Male	29	6	Leisure Activity Teacher 5
Focus Group 4 (Parents)	Male	34	5	Parent 1
	Female	48	30	Parent 2
	Female	45	21	Parent 3
	Female	46	20	Parent 4
	Female	51	33	Parent 5

The content analysis revealed three main themes (Table 3).

**Table 3:** Identified themes and subthemes

Theme	Categories	Subcategories
Health behaviors	Diet and nutrition	- Types of food consumed - Importance of a balanced diet for health - Barriers and facilitators to healthy eating - Common nutritional deficiencies in primary school children
	Physical activity	- Types of physical activity - Level and intensity of physical activity - Factors influencing physical activity - Barriers and facilitators to physical activity
	Sedentary behavior	- Negative health effects of excessive sedentary behavior - Types of sedentary behavior - Factors contributing to sedentary behavior in primary school children
	Mental health	- Stressors affecting children - Self-esteem and self concept - Barriers and facilitators to mental health
	Risk behaviors	- Substance use - Unsafe sexual behaviors - Physical aggression - Online risk behaviors
Environmental factors	Social influences	- Peer influence on behaviors - Family Influence on behaviors - School and community influence on behaviors
	Stakeholder collaboration and communication	- The role of community nurses in promoting healthy lifestyles - Inter-professional cooperation between teachers and leisure activity providers - Parent-teacher communication regarding children’s health-related behaviors
	Barriers and facilitators to promoting healthy behaviors	- Challenges for parents in promoting healthy behaviors at home - Environmental barriers - Individual barriers - Socioeconomic status - Media and technology - The role of leisure activities in promoting healthy behaviors
Education and empowerment	Health literacy and education	- Understanding health concepts - Access to health education resources - Importance of health education for behavior change

### Theme 1: Health Behaviors

#### Diet and Nutrition

Most of the FG participants reported that children today subsist on a very unhealthy diet consisting mainly of high-calorie or fast food, and that they perceive food not in terms of health, but mainly in terms of taste. There is a growing tendency among children of taking a superficial approach to eating instead of eating quality meals, which might be due to the increasing availability of a variety of unhealthy snacks. The FG participants were aware of the importance of a healthy diet and the need for specific foods (vegetables, fruits, carbohydrates, hot meals, etc.) in a diet. However, barriers to healthy eating, such as the unavailability and unaffordability of healthy foods, can make it difficult for children to maintain a balanced diet. Common nutritional deficiencies in primary school children include iron, calcium, and vitamin D, which can hinder growth and development.

*“Compared to a few years ago, their diets have become worse because there is too much fast food. Many teenagers spend money on fast food, not because they have to eat out, but because it is a way to socialize. It is also cheaper to buy unhealthy food than healthy food. Their diet contains few or no vegetables and they avoid red meat, fish and eggs.”* (Health Professional 1).

#### Physical Activity

With regard to the level of physical activity in primary school children, the FG participants noted that although children participate in a range of physical activities, including extracurricular activities and structured exercise programs in primary schools, their level of physical activity is too low. For some children, the amount and intensity of structured physical activity (e.g. sports activities at school) are also below the recommended levels. Children spend significant amounts of time engaged in sedentary activities such as watching TV, playing video games, and using the computer. Factors influencing children’s levels of physical activity include age, gender, socioeconomic status, parental support, school policies and environment, and access to safe outdoor areas for play and exercise.

*“Children’s sports education cards clearly show that their motor skills are declining. The hardest thing is to motivate them*.” (Leisure Activity Teacher 4).

#### Sedentary Behavior

The FG participants stated that sedentary behavior is a growing problem among children, with many children spending long periods of time in activities such as watching TV, using electronic devices, and playing video games. Excessive sitting leads to a rise in childhood obesity and chronic cardiovascular disease. Factors contributing to sedentary behavior in primary school children include the availability of electronic devices, social norms, and parental influence. Parents and teachers are increasingly aware of the negative effects of excessive sedentary behavior and encourage children to be physically active and spend less time on screens.

*“I’m really worried about how much time my child spends sitting, watching TV or playing video games. I know it’s easy if we let them do it, but I worry about the negative impact it has on their health and well-being. I would like to encourage them to be more active and find ways to break up the time they spend sitting. Today, even in schools, they are encouraged to do most of their work electronically or manage information that is not physically accessible.”* (Parent 4).

#### Mental Health

Most FG participants felt that children’s mental health faces a number of stressors that can affect their well-being, including learning pressures, social isolation, and family problems. Self-esteem and self-concept play an important role in children’s mental health, but tend to be negatively affected by social media and peer influences. Stigma and limited of access to mental health services were cited as barriers to positive mental health, while supportive relationships and access to resources were seen as its facilitators. The majority of respondents agreed that health professionals play an important role in promoting positive mental health in children and addressing mental health problems as they arise.

*“As a teacher, I find that children are stressed, especially due to exams and family problems. Social media has a big impact because it negatively affects their self-image.”* (Teacher 3).

#### Risk Behaviors

All FG participants agreed that the most common unhealthy behaviors among primary school pupils are alcohol consumption, tobacco smoking, and provocative clothing. However, the responses of the environment and especially adults who are important to the child (parents, teachers) to such risky behaviors are often inadequate or even counterproductive. Respondents also pointed to various forms of violence, ranging from harassment through social networks to physical and verbal aggression, which are very common among children.

*“I fear that our world is so advanced and dangerous for our children because all this access to different information can lead to a child developing unhealthy and dangerous habits or even tendencies. We simply cannot control it anymore; it seems to have gotten out of hand.*” (Health Professional 5).

### Theme 2: Environmental Factors

#### Social Influences

The impact of adult behavior on the promotion of a healthy lifestyle in children is of great importance. Through observation and imitation, children acquire crucial patterns of behavior and retain them throughout their lives. Upon identifying differences in FG participants’ opinions, attitudes, and habits concerning some of the factors that shape children’s lifestyles, the influence of peers was found to be substantial, as children often seek guidance and support from their friends. Children exposed to peers who engage in risky behaviors such as substance abuse or unprotected sex are more inclined to imitate such behaviors. Family dynamics play an influential role in shaping children’s behavior, with parents acting as the role models for healthy habits that promote healthy behaviors in children. In addition, low-income families often have more limited access to resources such as healthy food, safe neighborhoods, and quality health care, which can have a detrimental impact on children’s health and behavior. The school and community environment can also have a strong impact, with positive attitudes and supportive resources promoting healthy behavior.

*“Children are in trouble because they have no foundation, because they cannot always come first, because there is a lot of competition, even between parents: mine has to be better than yours. That’s why the child starts to compete with himself. But it seems to me that we put too much pressure on them, it seems to me that we limit them quite a lot, that we put quite a lot of pressure on them, and it’s not only us who do that.”* (Parent 2).

#### Stakeholder Collaboration and Communication

Collaboration between parents and educational institutions is of utmost importance for children’s development. In this section, FG participants explained how they perceived their role and the role of others in trying to build a successful and supportive relationship with their child.

They highlighted the integral role of nurses in promoting healthy behaviors through the provision of education, resources, and support to families and through cooperation with other health professionals. Inter-professional collaboration between teachers and leisure activity teachers can also have a positive effect on children’s lifestyles by promoting physical activity and healthy behaviors outside the classroom. Parents and teachers can work together to establish healthy routines and practices and address any concerns or challenges pertaining to children’s health-related behaviors.

*“It would be ideal for us to work together, say, by creating stronger links between the health and education sectors. Through various lectures for children, parents, and teachers. This responsibility should not rest solely on one sector. I think it would be most important to educate parents, and this should start right from the beginning, perhaps as early as maternity school.”* (Health Professional 4).

#### Barriers and Facilitators to Promoting Healthy Behaviors

Barriers and facilitators to promoting healthy behaviors refer to factors that can either hinder or encourage the adoption of healthy practices. Challenges for parents in promoting healthy behaviors at home include lack of time, conflicting priorities, and difficulties in implementing healthy habits. Environmental barriers such as limited access to healthy foods, neighborhood safety concerns, and limited access to safe spaces for physical activities can also hinder the adoption of healthy behaviors. The role of leisure activities in promoting healthy behaviors is also significant, as participation in organized sports and other physical activities can facilitate the development of healthy habits and social relationships. Moreover, the availability of community resources such as parks and recreational facilities can also contribute to the promotion of healthy behaviors. Individual-level barriers to healthy lifestyles among children include lack of motivation or interest in physical activity or healthy eating, inadequate knowledge or self-discipline, peer pressure, and underlying health conditions. One of the most significant barriers is the impact of modern technology on children’s behavior. Participants in the FGs noted that children today lead increasingly sedentary lifestyles and spend more time on screens than socializing or engaging in physical activity. Physical activity may be viewed as a low-priority activity, and low socioeconomic status can lead to limited access to healthy food, safe environments for physical activity, and quality health care.

### Theme 3: Education and Empowerment

#### Health Literacy and Education

Most FG participants stressed that health literacy and education have a significant impact on promoting healthy lifestyles among children. Understanding health concepts and access to health education resources are the key factors in promoting healthy behavioral changes as they enable parents and children to better understand the importance of healthy eating and regular physical activity. In addition, participants stressed the importance of integrating health education into the school curriculum and providing children and parents with access to quality health education resources tailored to their needs and understanding.

*“I think we have enough autonomy in education to include this in the curriculum, and the problem is on your side, because there should be more of you to implement this program. Ideally, the health professional should be present in schools all the time and pupils should have access to them for various problems.”* (Teacher 5).

## Discussion

The aim of the study was to identify the differences in children’s experiences and perceptions of their lifestyle among primary school teachers, community nurses, leisure activity teachers, and parents/carers, and to higlight their role in health promotion. The FG participants noted that children today have very unhealthy diets, low levels of physical activity, and discussed the barriers and risk factors that have a significant negative impact on children’s lifestyle. Parents consider risky behavior an important cause for concern. School is an important environment for health promotion, and health professionals should become more actively involved in the school environment through health promotion and education content, with the aim of training educators and parents. Other studies have come to similar conclusions that primary school children and adolescents experience problems related to poor eating habits, insufficient physical activity, unhealthy leisure time activities, and risk behaviors such as alcohol and tobacco use, which can lead to health problems [16, 18, 20, 22, 26, 33, 35, 36, 45, 51–53, 59, 63, 66, 69, 72, 76].

Unhealthy eating habits in children are linked to chronic diseases such as obesity, hypertension, high cholesterol, and diabetes [1]. The causes of unhealthy eating habits include marketing pressure from the food industry, media influences, peer pressure, changes in family dynamics, lack of time due to school and other commitments, and limited access to healthy food choices [1, 47]. Our study shows that primary school children often eat fast or industrially prepared foods, sweet and salty snacks, and do not consume enough vegetables or fruits. They lack the time and knowledge for food preparation, or are prevented from preparing food by their parents for fear of adverse events (burns, cuts, mess, etc.). Adolescents also struggle with unhealthy eating habits, such as inadequate vegetable and seafood intake and frequent consumption of fast food [26, 35, 53, 59]. They often eat only two meals a day and rely on nutrient-poor snacks that lack important vitamins and minerals [52, 66].

The physical performance of children and adolescents is affected by inadequate nutrition, resulting in a decline in their motor performance as reported by the participants in our study. While parents and the family environment play a pivotal role in meeting children’s needs for exercise and physical activity, lack of time resulting from a fast-paced life and overcrowded schedules hinders exercise. Environmental and social factors and overuse of modern technology also affect children’s physical activity. The Department of Health and Human Services [19] in the USA recommends moderate to vigorous physical activity for at least 60 min per day or three times per week. Sedentary lifestyle habits persist into adulthood and cause chronic non-communicable diseases [33], and in turn, insufficient leisure time physical activity increases health risks [15, 61]. Children are also among those most strongly affected by the COVID-19 preventive measures that have altered their lifestyles and could result in a lower educational attainment, higher morbidity from chronic non-communicable diseases, lower quality of life and lower work efficiency, affecting society’s economic competitiveness and potentially shortening life expectancy [11, 45].

Children and adolescents are a very vulnerable group who can quickly develop risk behaviors. Certain behaviors are particularly dangerous, such as long-term dieting, and young people are often not even sufficiently aware of the consequences of their actions [58]. In our study, alcohol and tobacco use were identified as two major issues affecting children’s health and well-being. In addition, perceptions of risk behaviors in the eyes of our respondents were also found to be associated with provocative clothing worn by girls. Risk behaviors can affect various dimensions of a child’s life, including their health, safety, autonomy, and freedom [76]. Parents play a crucial role in preventing risk behaviors in children and adolescents, as they can understand their child’s development and experiences better than anyone else and must adapt to the current cultural and technological landscape [22]. However, parents often justify their children’s risk behaviors with stereotypical arguments such as “everyone else does it”, “risk is part of growing up”, or by recalling their own youthful behavior, saying “I did that too when I was young”. Yet, both prohibitive and permissive parenting styles lack the arguments and critical information necessary to develop a coherent and knowledge-based approach to their growing children [50].

In our study, children’s mental health was found to be influenced by several factors such as social media and peer pressure, learning pressures, social isolation, and family problems, which can have a detrimental impact on children’s behavior and lifestyle choices. Poor mental health has been associated with increased sedentary behavior, poor nutrition, and insufficient physical activity [10, 63]. However, some studies suggest a positive relationship between mental health and healthy lifestyle: children with higher self-esteem and life satisfaction are more likely to engage in physical activity and consume healthy foods [42]. Accordingly, promoting a healthy lifestyle should prioritize children’s psychological well-being and include strategies such as exercise, nutritious meals, and supportive social environments [51].

The health of children and adolescents is influenced by a complex interplay of biopsychosocial, cultural, environmental, and economic factors. The causes of unhealthy lifestyle, inadequate nutrition, and lack of physical activity often lie in socio-demographic and economic conditions (level of educational attainment, employment, financial well-being) [25]. Parents play a vital role in ensuring healthy behaviors in their children. One of the greatest challenges is lack of time, as many parents juggle work and other commitments, leaving little time for planning healthy meals or physical activities. It is important that parents involve their children in meal planning and preparation, set aside time for physical activity, and model healthy behaviors themselves. Family support prepares young people to deal with stressful situations and protects them from the harmful effects of multiple negative influences [68]. Our research also shows that peers are valuable social contacts who contribute to young people’s health and well-being, but can also negatively influence risk behaviors such as smoking and drinking. Since the formation of a child’s identity depends heavily on feeling accepted in society, adolescents imitate their peers in order to be accepted [16, 72]. Our study identified several individual-level barriers that negatively affect children’s lifestyles, including lack of motivation and self-discipline, peer pressure, and the desire for instant gratification. Other studies have identified different individual-level barriers. For example, in a study by Saxena et al. [65], lack of knowledge and skills related to healthy lifestyles was found to be a major individual-level barrier in children.

The participants in our study associated socio-economic status with children’s behavior, health, life satisfaction, and body image. Children from wealthier families were perceived to be healthier, more satisfied with life, and displaying “external prestige”. In contrast, children from families with lower socio-economic status were perceived as having different perceptions regarding their health, life satisfaction, body image, amount of exercise, fruit consumption, frequency of injuries, and tooth brushing. Adolescents from more affluent families were more likely to have more health-conscious parents who were also more positive role models and led them to develop healthier lifestyles. Other research has shown that adolescents from less affluent and non-traditional families, and from families with non-working parents have poorer health, lead less healthy lifestyles, and engage in riskier behaviors than adolescents from more affluent families [12, 36]. Adolescents from families with higher socio-economic status have been found to engage in physical activity more often and tend to choose leisure activities that represent quality use of time compared to their peers from families of lower socio-economic status [55].

As highlighted by the participants in our study, rapid advances in modern technology and increased use of social networks and gaming in recent years have led to children and adolescents spending more and more time with technology. Children have moved from active leisure to passive screen time. Digital technology use is associated with higher levels of sedentary behavior and insufficient physical activity, leading to poorer psychophysical fitness and obesity [81]. Similar findings have also been reported by Dresp-Langley [21] who states that excessive use of digital technologies in children can lead to overweight, poor sleep, social isolation, poorer academic performance and even addiction. Nevertheless, the use of modern technology can also bring positive effects, as the Internet offers adolescents the opportunity to explore things that interest them on their own, while social media helps them form and maintain friendships [30]. However, the impact of screens on the development and health of children and adolescents depends not only on screen time, but also to a large extent on the content to which they are exposed, and the way and purpose for which they use their devices [21].

The participants in our study acknowledged that educational courses and programs now include more content on healthy living than ever before. Yet, they expressed concern about the lack of knowledge and support for teachers who feel overburdened with other responsibilities. Health professionals should therefore become more involved in schools to address this issue. While teachers are the key implementers of health promotion content, they lack the necessary knowledge and skills and rely heavily on online resources. Many teachers feel that nurses should take a more active role in health education and address topics such as first aid, sex education, mental health, and personal hygiene. Training courses for parents, teachers, and health professionals can help raise healthy individuals who value their health [18, 40, 41, 64]. More content and programs are needed to address health-related behaviors and highlight the importance of health education within families [45].

The collaboration between teachers and leisure activities teachers, can immensely benefit children. By working together, they can provide youngsters with a variety of physical activities that promote healthy lifestyle choices and foster knowledge about nutrition while developing valuable habits [4]. Similarly, parent–teacher communication about children’s health behaviors can raise parents’ awareness of their child’s health status and encourage them to work with teachers to support healthy behaviors both at home and in the classroom [5]. By sharing information about a child’s health and well-being, parents and teachers can collaborate in promoting healthy eating habits, regular physical activity, and adequate sleep. It is important to note that effective communication and collaboration between these groups of stakeholders requires mutual respect, trust, and understanding [8]. Schools are viewed as an appropriate setting for promoting healthy lifestyles due to the time children spend there, the infrastructure they offer, and the role they play in education and community health [20, 43]. Teachers play a major role in health promotion as they accompany children every day, and healthy children show higher learning capacities and attendance rates [18, 49]. Health promotion also leads to lower absenteeism and positive academic outcomes, while active promotion sets a positive example and helps prevent risk behaviors.

Undeniably, health education is immensely important in primary schools. Its significance lies in its ability to encourage healthy habits while reducing the risk factors of chronic diseases among children. Research has substantiated that when children are equipped with this knowledge, they are more inclined to adopting behaviors such as physical activity, healthy eating habits or seeking medical care whenever it is required [32]. In addition to fostering critical thinking and problem-solving skills, participation in this educational program increases one’s confidence and proficiency towards their overall wellbeing [79]. However, access to health education is limited in low-income or rural communities, leading to disparities in health-related knowledge and behaviors [62]. It is therefore crucial to ensure that all children have access to health education resources. Studies demonstrate the importance of health education for behavioral changes, improving knowledge of nutrition, hygiene, and healthy behaviors, and reducing rates of obesity and chronic disease [14, 80].

### Implications

It is imperative to ensure that primary school curricula incorporate or reinforce health-related content, and provide school staff and parents with adequate training. This will enable schools to create a supportive environment that promotes healthy behaviors and lifestyles among children. By integrating health education into the curriculum, schools can help children develop healthy habits and attitudes to nutrition, exercise, and other lifestyle factors. Providing adequate training to school staff and parents will also ensure that they possess the knowledge and skills to support children in developing healthy behaviors and making informed choices about their health. One way society can guarantee its future success is by promoting adequate health education and providing thorough training for young people. With such provisions, positive attitudes toward healthier lifestyles will undoubtedly manifest themselves over time, leading to an upsurge in the quality of life amongst this demographic.

### Limitations

The potential limitations of qualitative methodology primarily revolve around the inability to generalize beyond the study’s sample, and the potential subjective influences of the researcher. We attempted to mitigate these limitations by incorporating certain measures already in the research design stage (e.g., planning/structuring the main questions). In addition, it is important to acknowledge the cultural context of the study and the geographically limited rural area, which may hold a different perspective on lifestyle choices than an urban area. Therefore, future research should focus on a sample of all those involved in educating today’s generations of primary school children from different regions and communities to provide a more comprehensive insight into different aspects of children’s lifestyles.

A systematic inclusion of an additional health education subject in the primary school curriculum for pupils, teachers, and parents would be an appropriate measure. The findings of our research can serve as the foundation for developing an individualized, evidence-based model of health promotion at the local level and set an example of best practice for other municipalities.

## Conclusions

The results of our study demonstrate a concerning rise in risk behaviors and unhealthy habits among children, highlighting the crucial responsibility of parents, teachers, peers, and health professionals in promoting healthy habits. Children may resort to unhealthy habits and substances, such as violence and fast food, when left to make independent decisions. Early education on healthy habits and attitudes is imperative for the development of healthy behaviors and attitudes towards health. The participants in this study emphasized the need for collaboration among all stakeholders involved in children’s education to raise awareness, promote positive behavioral patterns, and identify risk behaviors. Further research is required to develop effective strategies to prevent chronic diseases and empower children to pursue healthier lifestyles.

## Electronic Supplementary Material

Below is the link to the electronic supplementary material.

Supplementary Material 1
